# The experiences of new mothers accessing feeding support for infants with down syndrome during the COVID-19 pandemic

**DOI:** 10.1080/20473869.2022.2109000

**Published:** 2022-08-14

**Authors:** L. Hielscher, A. Ludlow, S. E. Mengoni, S. Rogers, K. Irvine

**Affiliations:** 1 Department of Psychology, Sport and Geography, University of Hertfordshire, Hatfield, UK; 2 University of Hertfordshire, Hatfield, Herts, UK

**Keywords:** Down syndrome, feeding problems, COVID-19

## Abstract

Infants with Down syndrome are more likely to experience feeding problems and mothers are likely to require more feeding support than mothers of typically developing infants. During the COVID-19 pandemic, many feeding support services changed from face-to-face to online, which impacted some maternal feeding experiences negatively, but no studies to date have explored the impact for mothers of infants with Down syndrome. Thematic analysis was conducted on semi-structured interviews from thirteen new mothers of infants (aged 8–17 months) with Down syndrome in the UK. Three superordinate themes were generated: (1) Every baby with Down syndrome has a unique journey, (2) There’s no point asking, they won’t know, (3) Lack of in-person support. Many mothers expressed frustrations over health professionals’ lack of Down syndrome specific knowledge resulting in unmet needs, further magnified due to the nature of the virtual support being offered. Moreover, mothers struggled with reduced social support from other mothers when encountering feeding problems. These results hold real-world implications for health professionals who could provide more specialised face-to-face feeding support to mothers of infants with Down syndrome. This should be prioritised for children’s overall development and mothers’ wellbeing.

## Introduction

Down syndrome is the most common cause of genetic intellectual disability with a high prevalence rate of 1.0 to 1.5 per 1,000 births (Strippoli *et al*. 2019), and it is generally diagnosed at or before birth. As highlighted recently by the Center for Disease Control and Prevention, health professional services that are made available early in life are crucial for fostering the physical and intellectual abilities of infants and children with Down syndrome (CDC 2021).

Feeding problems are common in childhood, including dysphagia, difficulty latching successfully and food refusal, occurring in 80% of those with developmental delay compared to 25–35% of typically developing children (Machado *et al*. 2016, Anil *et al*. 2019). Specifically, individuals with Down syndrome present with a variety of issues which can negatively impact feeding (Anil *et al*. 2019). For example, attainment of feeding milestones such as introduction of solid foods and development of self-feeding skills can occur 10–35% later in infants with Down syndrome (Nordstrøm *et al*. 2020). Additionally, infants with Down syndrome often present with hypotonia, which can lead to difficulty with positioning during breast and bottle feeding, poor lip seal, difficulty sucking, inefficient swallow leading to choking and difficulty sitting upright when introducing solid foods (Agostini *et al*. 2021).

Difficulties with sensory processing, akin to that found in other developmental disorders, can lead to behavioural feeding problems such as food refusal, selectivity by type and texture and refusal to swallow (Field *et al*. 2003). Furthermore, a variety of physical factors also affect feeding concerns in infants with Down syndrome; for example, 40–60% of infants born with Down syndrome have a congenital cardiac anomaly which can lead to daily tasks being more exhausting (Marder *et al*. 2015). To cope with fatigue, infants with Down syndrome may feed more frequently and for shorter durations and early feeding milestones may be disrupted (Pisacane *et al*. 2003). Moreover, anatomical differences such as a small oral cavity, large tongue, and short palate can also result in feeding problems such as tongue thrust, abnormal tongue movement and pocketing of food, sneezing and choking (Ooka *et al*. 2012).

Feeding problems can be difficult to manage and it is important that parents are supported to manage them (Estrem *et al*. 2016). For example, research has consistently shown that the quality of feeding support for new mothers is important for good maternal mental health (Chaput *et al.* 2016). Poor feeding support is associated with reduced breastfeeding duration and increased risk of post-natal depression symptoms amongst new mothers (McFadden *et al*. 2017). Furthermore, the risk of depression, grief and trauma increases when mothers end breastfeeding earlier than planned due to the difficulties encountered (Brown and Shenker 2021). Lack of early feeding support can have consequences for the infant, including low dietary variety, inadequate daily total energy intake, limited weight gain and increased duration of mealtimes (Hopman *et al*. 1998, Lewis and Kritzinger 2004).

Research conducted in the UK before the pandemic highlighted the feeding support available for mothers of infants with Down syndrome was already inadequate (Cartwright and Boath 2018) and families of infants with Down syndrome were more likely to report unmet care needs (McGrath *et al*. 2011), including a need for more face-to-face contact (Sooben 2010). Colon *et al*. (2009) found a third of mothers of infants with Down syndrome in their study reported receiving no feeding support and health professionals were unable to advise on specific feeding problems related to Down syndrome. Furthermore, where professionals lacked Down syndrome specific information, they were unable to refer mothers for guidance elsewhere (Cartwright and Boath 2018).

As a result of the COVID-19 pandemic and subsequent lockdowns that occurred during this time, the provision of National Health Service (NHS) feeding support services in the UK have changed. This is not unique to the UK, a recent international survey found that 74% of parents of children with intellectual and developmental disabilities reported that their child lost access to at least one therapy or education service, and 36% of respondents lost access to a healthcare provider (Jeste *et al*. 2020). In some regions, services have been reduced and staff re-deployed to other areas, and some services have been delivered in a new format (e.g. over the phone, video call) as opposed to the traditional face-to-face mode of delivery (Brown and Shenker 2021).

Uncertainty remains over the effectiveness of feeding support delivered using a virtual format (Coxon *et al*. 2020), with the move to online support reported to have impacted maternal feeding experiences negatively (Vazquez-Vazquez *et al.* 2021). For example, Vazquez-Vazquez and colleagues found that 45% of mothers who gave birth during lockdown felt they had received inadequate support with feeding their infant. Additionally, 57% of mothers who had given birth prior to lockdown reported a reduction in the infant feeding support they consequently received during lockdown.

The emotional support that face-to-face interactions can facilitate is another important element of post-natal care (Schmied and Lupton 2001), with a lack of empathy from health professionals shown to increase new mothers’ hesitancy to ask for practical support when needed (Fox *et al*. 2015). Importantly Brown and Shenker (2021) reported that 36% of participants they surveyed felt they had not received enough emotional support from health professionals during the pandemic. Moreover, 70.3% of these mothers citied the lack of available professional face-to-face feeding support as the main reason for stopping breastfeeding earlier than planned.

To date, there has been a paucity of research presenting mothers’ voices and lived experiences of feeding infants with Down syndrome. Moreover, there is a need to examine how COVID-19-related changes to feeding support services have affected this group of mothers who reported struggling to access sufficient support prior to the pandemic. To address this need, mothers of infants with Down syndrome who gave birth shortly before and during lockdown took part in semi-structured interviews.

This study aimed to identify the needs and personal experiences of mothers accessing feeding support for their infants with Down syndrome and how this may have been impacted by the COVID-19 pandemic.

## Materials and methods

Of the little existing research which has examined feeding infants with Down syndrome, the vast majority has adopted a quantitative approach (Cartwright and Boath 2018). Therefore, a qualitative approach was selected for the present study to allow exploration of mothers’ experiences of accessing feeding support in rich detail. Participants took part in semi-structured interviews and then reflexive thematic analysis (Braun and Clarke 2006, 2019) was conducted to explore and identify common themes amongst the experiences of participants.

To be able to consider the interview findings in the context of service-related changes, a questionnaire was first developed and distributed via email to various feeding support services in the local county and surrounding areas. This included hospital-based infant feeding and maternity units, health visitors, family centres and NHS community trusts. Around 20 services were contacted by email to distribute the survey. To encourage busy services to complete the questionnaire, they were provided with an overview of what the questionnaire aimed to explore and encouraged to select a relevant individual to complete the questionnaire, on behalf of the service. The questionnaire aimed to gather information about how feeding support delivery has changed since the beginning of lockdown in March 2020, up until the time the questionnaire was distributed (June and July 2021). The questionnaire was not targeted at individuals with specific role titles. The questionnaire was shared with a variety of organisations whereby people with many different role titles had the opportunity to comment on its operation throughout the pandemic. Furthermore, throughout the pandemic, many health professionals were re-deployed to other departments and thus were working in environments that may have been incongruent with their official role title.

For the qualitative interviews with the parents, a demographic questionnaire was completed and experiences were explored through interviews. The semi-structured interview schedule aimed to explore mothers’ experiences of feeding and feeding support both in hospital immediately after birth and in the community. To ensure that the interview schedule was suitably sensitive and appropriate, this was developed and then refined following discussions with the research team and a parent of a young person with Down syndrome who also has professional experience in health and education for young children with Down syndrome.

Key questions included:

- Can you describe your experience of feeding your baby shortly after birth?
- Can you describe the support you received with feeding your baby whilst in hospital?
- Has lockdown impacted your experience of feeding your child in any way?

Due to the social distancing measures in place at the time, video interviews were conducted through an online platform (Microsoft Teams). Following the interview, participants were given the opportunity to share further information, thanked for their time and were given a debrief sheet. Recordings were made of each interview and were transcribed verbatim by the lead author.

### Participants

A purposive sampling method was used to recruit 13 mothers of infants with Down syndrome in the UK who gave birth either in the 12 months prior to the beginning of the first UK lockdown (23rd March 2020) or during lockdown. To be eligible for inclusion in the study, mothers must have been at least 18 years of age, and able to speak fluent English, without assistance. Individuals were excluded for participation in the study if their child was born before 23rd March 2019, and if they did not have a child with a diagnosis of Down syndrome. It is worth noting that whilst the first UK lockdown began a phased ending from 4th July 2020, a variety of restrictions were still in place throughout the majority of 2020 and further lockdowns occurred in the latter part of 2020 and 2021. As such, women in the sample who gave birth after the first ‘full’ lockdown would have still been impacted by restrictions and social distancing measures related to COVID-19. Recruitment specifically focussed on mothers as opposed to parents because attendance at health settings was often restricted due to COVID safety measures. As a result, mothers may be likely to have had a greater number of experiences related to feeding and accessing feeding support, especially in the immediate post-natal period.

To recruit participants, information about the study was shared via websites and social media groups used by mothers of infants with Down syndrome. This included infant feeding and mother and baby support groups on social media. Information about the study was also shared via existing contacts with relevant local professional and support organisations and groups, and via word of mouth.

Mothers were invited to contact the research team via email to volunteer to take part in the study. They were provided with a digital participant information sheet and consent form. Mothers provided their consent to participate by completing and signing the consent form and then emailing the digital document (or a scan of the hand completed version) back to the researcher.

The concept of information power was used to guide our sample size (Malterud *et al*. 2016), with the concept that the more relevant information a sample holds, the fewer participants are needed. This approach has been advised for researchers using a reflexive thematic framework (Sim *et al*. 2018a, Braun and Clarke 2021)

### Ethical considerations

Approval was given by the University of Hertfordshire Ethics Committee (approved protocol number: LMS/PGT/UH/04532) and this study was conducted in accordance with the Declaration of Helsinki. Participants were provided with sufficient information about the study to facilitate providing their informed consent. Participants were made aware that they would be recorded and how the recordings would be saved, stored and deleted upon transcription. Confidentiality was adhered to throughout the study. During transcription, identifying details were removed and pseudonyms applied. All names used throughout this report are pseudonyms (including names that are referenced in quotes from participant interviews). Participants were provided with a debrief sheet including supportive websites following the interview.

### Data analysis

Reflexive thematic analysis was used to develop an understanding and interpretation of participants’ subjective experiences, and identification of common themes amongst them (Clarke and Braun 2018). The lead author read and listened to the data several times to ensure familiarity; and then began line by line coding of the interview transcripts, noting emerging themes, comments and interpretations. Transcripts were analysed primarily on a semantic level (i.e. ideas and explanations explicitly communicated by the participants were prioritised). Emerging themes were collated across transcripts and analysed for similarities, creating subordinate themes across the data. Through discussion with the research team, subordinate themes were refined and analysed, and further grouped under superordinate themes. To ensure rigour and credibility in the data the final themes table with supporting quotes was developed through supervision; member checks were completed by giving all participants access to the finalised table for comment.

## Results

In order to provide context for the results of the thematic analysis, the results of the questionnaire given to feeding support services are first presented (Table 1). Despite efforts to distribute the service questionnaire as widely as possible, response rates were very low. Eleven responses were received, but not all participants answered every question. Two responses were from NHS trusts and four were from family centres. Five respondents did not state their organisation.

**Table 1.** Results from questionnaire distributed to professionals working in feeding services.

Comments regarding changes to services during the pandemic	No. of services this change was reported by	Positive feedback from staff and their service users about feeding support services during the pandemic	Negative feedback from staff and service users about feeding support services during the pandemic
Feeding support services were stopped or changed during the pandemic, including peer support in hospital, drop-in breastfeeding clinics and face-to-face introduction to solids seminars at family centres. Some of these services remained available face-to-face but by appointment only instead of as a drop-in service and some sessions were offered via video call instead. Circumstances under which face-to-face appointments went ahead were where there were safeguarding concerns, where a full feeding assessment was required (e.g. in cases of a potential tongue tie), and if virtual consultations had not addressed parents’ needs).	5	A switch to video and telephone appointments in some services meant that more service users could be contacted. Beneficial for staff to see where parents usually sit and feed baby at home. One service conducted an infant feeding survey and found that 93% of families surveyed were happy with the virtual drop-in service.	Two staff members from different organisations felt that feeding support availability at their service had reduced in this time. Some service users were unsure where to access feeding support. Staff confusion over service availability meant that staff were not referring service users to tongue tie clinics or Health Visiting specialist clinics, mistakenly thinking that they were not running anymore. Number of different options of support available to mothers was reduced. One service received feedback that mothers would like more universal drop in feeding groups that anyone could attend (which were available pre-pandemic), as opposed to specific groups for people with certain difficulties.
Some existing feeding support services increased throughout the pandemic.	3	One staff member felt that the overall quality of feeding support their service offered had improved throughout the pandemic.	
No feeding support services were increased throughout the pandemic.	3		
Introduced new 1:1 and group support sessions via telephone and video cal e.g. new appointment at 3 months old	4	Being able to access support in service users’ own homes was very positive. New systems were time effective. Video calls were beneficial for staff—allowed a closer view of baby’s feeding than would have been possible from in-person appointment where a 2 m distance would have been required.	
		Service users were happy with extra contact at three months	

**Figure 1.**
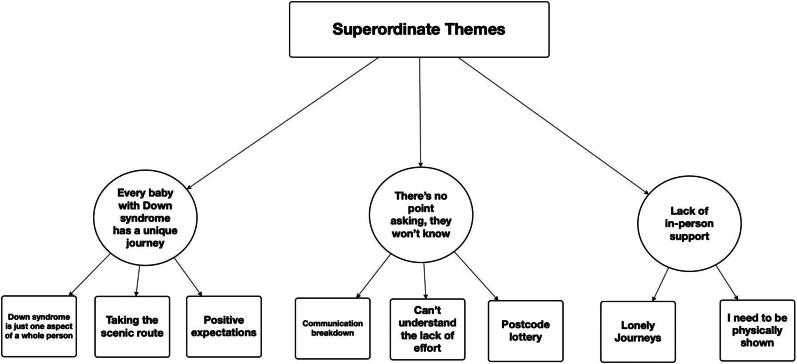
Superordinate themes and their sub-themes.

Analysis of thirteen semi-structured interviews with mothers produced three superordinate themes; (1) *Every baby with Down syndrome has a unique journey*, (2) *There’s no point asking, they won’t know*, and (3) *Lack of in-person support* (Figure 1). Interviews lasted between 39:31 and 69:01 min (*M =* 54:55, *SD =* 8:50). Descriptive information about the overall sample is presented in Table 2 and characteristics of individual participants and their infants can be seen in Table 3. One participant (Kaisha) did not return the demographic details form (and so Table 3 does not include her data) but some demographic details were provided through her interview.

**Table 2.** Sample characteristics.

	Maternal characteristics	Infant characteristics
Mean age	35.5 years, ***SD***= 3.9	11.8 months, ***SD***= 3.0
Age range	28–40 years	8–17 months
No. who gave birth before lockdown	3	
No. who gave birth after start of first lockdown	9	
No. of the sample that were first time mothers	6	
Postnatal hospital stay duration (range)	1–15	1–35
Postnatal hospital stay duration (mean)	6, ***SD***= 5.0	9.7, ***SD***= 9.9

**Table 3.** Characteristics of individual participants and their infants.

Participant Pseudonym	Age (years)	Country of residence	First time mother?	Child born before or after start of 1st UK lockdown	Mother’s postnatal hospital stay duration (days)	Automatic referrals to relevant services^1^ when leaving hospital?	Child age (months)	Infant’s postnatal hospital stay duration (days)
Megan	39	England	Yes	Before	14	No	16	35
Amber	38	Scotland	No	After	4	No	11	4
Christine	28	England	Yes	After	12	No	8	12
Lily	34	England	No	After	1	No	9	1
Nicole	34	England	Yes	Before	15	No	16	15
Robin	29	England	Yes	After	5	Yes	13	20
Jen	37	England	No	After	2	No	10	2
May	40	England	No	After	2	No	9	2
Evie	36	England	Yes	After	2	No	12	2
Claudia	38	England	No	After	7	No	10	7
Isabelle	39	Northern Ireland	No	Before	6	Yes	17	6
Zena	34	England	Yes	After	2	Yes	11	10
Kaisha	–	England	No		–	No		–

^1^ This encompasses all support services relevant to infants with Down syndrome e.g. Speech and Language, Occupational Therapy, Physiotherapy.

### Every baby with down syndrome has a unique journey

This theme deals with the negative assumptions and expectations that can come with a diagnosis of Down syndrome, rather than acknowledging that every infant with Down syndrome has unique needs and strengths.

#### Down syndrome is just one aspect of a whole person

Seven mothers commented that they didn’t want health professionals to assume what their child’s feeding abilities and developmental trajectory would be based on blanket assumptions about Down syndrome, as illustrated by Lily:

So we had heart surgery and then obviously put an NG tube in 'cause obviously that's how she needs to be fed 'cause she's ventilated and everything else. But several nurses were like “oh, so she's tube fed at home?” Like it was an assumption that, well, she has to be tube fed because she's got Down syndrome. Whereas actually I was like no. She's bottle fed at home, and they were like, “really? I'm almost shocked and taken aback that she’s bottle fed.”

Many mothers had a strong feeling that infants with Down syndrome have differing strengths and challenges despite having the same diagnosis and so infants should be treated as individuals and their own personal strengths and needs explored.

What Jess can do physically, some of my friends’ little ones can’t, but they're way ahead of her in speech. You know, they're all so different and unique in their own little ways. They've all got some weaknesses as have we all, but I do think there is a lot of work to be done generally around information with feeding little ones with Down syndrome and there needs to be this sense of encouragement rather than this sense of I wouldn't even bother because they'll struggle. (Nicole)

Six mothers felt it was sometimes forgotten that their child is a baby first and foremost and that typically developing infants have feeding problems too. ‘The Down syndrome’ is not always the problem as illustrated by Kaisha, ‘For me with Thomas, I never really felt that the Down syndrome affected his feeding, at least not his milk’.

There were mixed impressions regarding experiences with health care professionals with Lily having a favourable impression: ‘The first thing she was just like, oh, isn't she gorgeous’. Whereas Amber has a very negative experience through this interaction.

She (health visitor) wrote in that red book that Emily suffered from Down syndrome. And I was like no she doesn’t. You know, just something like that. That seems so nothingy. It’s just a massive thing. She doesn't suffer, people with Down syndrome don't suffer because they have Down syndrome.

#### Taking the scenic route

Seven mothers in the sample reported feeling as if negative expectations were automatically placed on their child’s feeding abilities as a result of their diagnosis.

There were a couple of general midwives on the normal ward who actually said, I wouldn't even attempt to try and combi feed because it can be confusing for normal babies at the best of times. To which I thought OK. Normal babies, mine must be some kind of alien. (Nicole)

However, nine mothers felt that their infants would be just as able to reach a lot of the developmental milestones that typically developing infants do—they just may need different levels of support and time.

I get ahead of myself and I start to get really stressed and worried about it and I’m like, oh, but he should be, you know, eating fish fingers by now or whatever and it’s important for me to keep catching myself. He just needs a little bit more time (Evie)

Just because she has Down syndrome, it shouldn’t mean that she’s held back from doing things. You know, it might take her a little longer and that’s when, you know, they started describing things as taking the scenic route, which I think is quite a nice way of looking at it, really…. (Nicole)

#### Positive expectations

Six women noted how important it was to be given positive expectations of their child when coming to terms with a diagnosis or when struggling with feeding. Knowing it *was* possible for infants with Down syndrome to feed successfully helped them to better cope with challenges and to persevere.

I think even just having that knowledge that just because it's hard doesn't mean it's impossible. It just means that it's taking longer, can massively impact how you feel when it's happening. Because, if you don't know that, and it's hard, it was so easy just to feel like, oh, that means it just can't happen and I failed, it's just not working and everything is awful. (Evie)

This is especially pertinent because six mothers in the sample commented that the realities of breastfeeding were unexpected. As noted by Christine ‘I was so like, yes, I definitely want to breastfeed, it's been a lot more difficult than I thought’.

Three mothers also noted the impact that positive information about the lives, developmental trajectories and abilities of people with Down syndrome had on their ability to come to terms with the diagnosis and wishing they had this information at the hospital.

I mean, it's not necessarily like they were negative. But everything that I've learned about how amazing their lives can be and what you know how fulfilling they can be. And like you say, they can do whatever they want to. They just might need a little bit of extra support to get there. (Evie)

### There’s no point asking, they won’t know

This theme addresses the relationship breakdown between mothers and health professionals resulting from a lack of trust.

#### Communication breakdown

Eight mothers felt disappointed and lost faith in health professionals working with them and became reluctant to seek their support.

I think any trust with professionals had gone by that point. So I think I felt there's not much point trying to seek out anybody here, so we'll go online and find out what other people are doing. (Lily)

Instead, 11 mothers cited online resources and charities such as Positive About Down Syndrome (PADS) and the Down Syndrome Association (DSA) as a safety net in the face of their unmet needs, explaining that they didn’t know how they would have coped without online resources which they often turned to instead of asking health professionals. As stated by Christine ‘You know there's all these things and like I said, everything I've learned about Down syndrome. I've learned online through my own doing, not from anyone’.

I would use the Internet, not any of the professionals that dealt with us, I would ask the woman who runs Positive about Down syndrome, and she's obviously just a volunteer. (Lily)

Five of the mothers who had breastfed before felt more prepared to deal with the challenges and could rely on their ‘gut instinct’ making them less vulnerable and reliant on health professionals.

If I had been a first time mum I wouldn’t have felt confident and I would have doubted my own ability. I would have probably gone straight over to formula; I would have just given up with the breastfeeding. But because I’d breastfed two babies before I knew. (Isabelle)

#### Can’t understand the lack of effort

It was commonly reported that health professionals lacked knowledge around various elements of feeding infants with Down syndrome, leaving mothers to try and seek necessary support elsewhere.

Nobody that we spoke to knew much about Down syndrome. There was no specialist person to speak to who had knowledge around it, and so we were just left to flounder a little bit, we got sent home, told to Google. (Lily)

Five mothers reported that health professionals couldn’t signpost them to other services where they lacked knowledge of the issues themselves and questioned why the health professionals were not better informed about Down syndrome. This made mothers feel like health professionals didn’t care about supporting them.

Down syndrome isn't rare, they should have that knowledge there and actually when you're going into homes and working with mums that are trying to breastfeed babies or go into different appointments that they should know or they should be able to find out very quickly and easily to help you and support you and that was just never there. (Amber)

Three mothers found that professionals sometimes refused to acknowledge the diagnosis. For example, Amber stated ‘But you just think that because people don't understand or have a lot of knowledge so instead of asking they just don't ask and don't mention it’.

Mothers were unsure whether some of their feeding problems were directly a consequence of Down syndrome specifically and felt let down by professionals who shied away from mentioning it.

I think probably if there had been some more information towards feeding with Down syndrome. Because I thought all the feeding was due to him being premature and it's not since I've come out and I've read obviously everyone else's stories online that I realised things that I didn't necessarily realise at the time, I just thought he was so small. (Christine)

This sometimes presented as a reluctance to help and mothers such as Nicole and Evie were left feeling let down and abandoned with their problems:

I feel like they could have said yeah, that's the right position or just tweak it a little bit and move her around a bit more this way and you'll get it. That was all the advice that I was looking for.?…. Why is it that Down syndrome conjures up such negative connotations in people’s minds for them to think I don't think I can help this lady? (Nicole)

Kindness and support, I think that was really lacking and not in all of the interactions. Some of the midwives in the hospital were amazing, but it needs to be all of them. (Evie)

In contrast, Zena and May showed how positive the experience could be when health professionals were there to provide needed support.

They (NICU staff) were absolutely fantastic and I can’t fault the level of support that we did get, even from the consultants—they were absolutely fantastic. It was a very supportive environment actually. (Zena)

Having someone to talk through it with and someone who, that's their job. It felt a little bit more like, OK, I **can** cope with this because at times, especially in the middle of the night, it did feel a little bit like I'm not sure I can cope. (May)

#### Postcode lottery

There was an overwhelming feeling amongst mothers that the ability to access the appropriate level of care for their child’s needs shouldn’t be down to luck and you should be able to access the same services—regardless of geographic location.

It’s a postcode lottery and that’s wrong. it should be one service, it should be one size fits all…We know that a child with Trisomy 21 has development delays, so why wait until you see what those delays are? (Isabelle)

The level of support received by each mother differed considerably in terms of how much access they had to health professionals as they left hospital.

Well, we were quite lucky. I've since realised in our area we just got referred to literally everybody the day we left hospital. So, our consultant’s been brilliant and he just referred us to everyone, so he referred us straight to speech and language anyway. (Robin)

Mothers were aware of the variability in care access and expressed a feeling of having been lucky in receiving good support.

We could have easily gone home and not known that these are the type of people that need to be involved with her care, but they did it all for us and I couldn’t be more thankful. I think it’s unheard of, I don’t think a lot of people get that. (Zena)

If I didn’t have the speech and language therapist input, I probably wouldn’t have known about the chewing [issue], I have to feel for parents that don’t have that input. (Isabelle)

### Lack of in-person support

This theme explores the unmet needs of the mothers in the sample which resulted from reduced in-person contact due to social distancing measures.

#### I need to be physically shown

Four mothers felt that the virtual feeding support offered in place of face-to-face appointments was not effective. Many mothers who expressed they lacked confidence with positioning were not completely reassured and worried that professionals hadn’t been able to see what they were doing properly over video call.

You know, reading stuff in books and being told what to do isn't as useful as being shown what to do and doing it in person, and so just having that face to face did help. (Evie)

First time mothers found it difficult to describe elements of feeding that they were new to and didn’t understand to someone else as demonstrated by Robin:

The breastfeeding people rang me and sort of discussed over the phone, which wasn't massively useful because they're sort of saying oh are you trying this and like are you feeding him now and like what's he doing and I’m like, I don't know. He's he's, he's latched, maybe I don't know. It's hard to describe something that you don't really know what you’re doing down the phone to someone. (Robin)

Where feeding support services became exclusively virtual for some, eleven mothers expressed feelings of abandonment and helplessness.

I ended up with post-natal depression because I just felt so low with it and overwhelmed and that feeling of well, if you're not gonna help me, and you're not gonna help me, who is there to help me kind of thing? And then obviously the lockdown hit and it made it 10 times worse. (Nicole)

I've had quite a lot of health professionals employed by different people, some of them could come into my home, some of them can't. Some of them still can't. Some of them can meet me outside, and so it varied massively so and I know across the country it's varied massively, so some people across the country would have had less support than other people as well, which isn't ideal. (Robin)

#### Lonely journeys

Eight mothers struggled with reduced peer support due to social distancing measures, and craved reassurance and encouragement and reflected on how much easier this would have made it to persevere in the face of feeding problems. For example, Lily highlighted the lack of emotional support.

There was never anybody to call when you're in a bit of a panic in the middle of the night or in the morning, I just thought we didn't really have a person to turn to. (Lily)

It was almost moral support more than anything kind of practical advice. But I mean obviously practical advice would also have been really helpful, but having somebody there to be like don't worry, try again. (Nicole)

Eight mothers reported feeling lonely and isolated without the comfort of friends or breastfeeding clinics and expressed a deep desire to be around others who understood or were encountering similar challenges.

I didn’t realise first time around but what I needed was a support group and a group of women who were doing exactly the same as me and had the same issues and you know could give me advice. Or, you know, just people to talk to while you're feeding, even you know, kind of have a chat and a piece of cake or whatever. And that was amazingly useful for my mental health. (May)

## Discussion

The experiences of mothers of infants with Down syndrome who gave birth before and during the first UK lockdown were explored, to identify their personal experiences of feeding support and any resulting impact of the COVID-19 pandemic. The findings highlighted mothers’ desires for health professionals to view their child as an individual person, as opposed to just a diagnostic label, for the discourse to be more positive and to be supported if/and when breast feeding concerns arose. The findings highlighted a lack of face-to-face support received during the pandemic, which had a negative impact on mothers’ perception of the quality of support they received.

Mothers reported that too often the health professionals assessed their child’s abilities based on blanket assumptions about Down syndrome, instead of acknowledging that all infants have unique abilities. These mothers expected their children to reach the same feeding milestones as typically developing children but recognised that their journey to that point and support required along the way would look different. Therefore, there is a need for training of health professionals to highlight that although infants with Down syndrome may experience more difficulties breastfeeding, this should not rule it out as an option (Zhen *et al*. 2021). For example, Sooben (2012) acknowledges the importance of identifying individual feeding abilities and support requirements for both mother and child to promote successful breastfeeding. Where this is done effectively, infants with Down syndrome can go on to breastfeed successfully. Furthermore, feeling adequately supported can have a positive impact on the overall feeding experience and mental wellbeing of mothers as demonstrated by Zena, Isabelle and May.

Mothers highlighted the variability across feeding services for Down syndrome, such as differences in levels of knowledge of Down syndrome that existed amongst health professionals, leading to a disparity in care being accessed. Many expressed the desire for all health professionals to be better informed to support parents (Douglas *et al*. 2016). Similar to the findings of Cartwright and Boath (2018), parents expressed frustration at health professionals who avoided giving advice (despite mothers asking for support) for fear of saying the incorrect thing, feeling that health professionals were ‘out of their depth’. In the present study, this phenomenon left mothers feeling abandoned and also more heavily reliant on the Internet as a source of information about feeding in Down syndrome.

Additionally, it is interesting to note that the majority of mothers’ who participated in the study gave birth after the first lockdown. It is possible that more women who gave birth after lockdown volunteered to participate in the study because feeding support was a current or more recent concern and priority, given that their children were younger.

Moreover, the online delivery of feeding services removed the opportunities for mothers to meet other new mothers, yet social support from other women at breastfeeding groups has been reported to be more beneficial than professional feeding assistance by some mothers and can contribute to longer breastfeeding durations (Fox *et al*. 2015). Limiting face-to-face medical appointments in favour of virtual delivery can also inhibit the relationship that mothers develop with their midwives by preventing things like a comforting touch (Coxon *et al*. 2020). On the other hand, recent research suggests that virtual feeding support can be beneficial for many mothers and telehealth services can be more convenient and easily accessible than face to face support for some (Feinstein *et al*. 2021). This may be particularly applicable to those who may find it difficult to travel to in-person appointments or groups e.g. in the early postpartum period, or mothers with multiple children . Some groups (for example first time mothers and/or mothers of infants with more feeding problems) may desire more face-to-face support and our research suggests that the provision of face-to-face support should be prioritised for these groups. It is important that mothers are given options regarding their care and the delivery of support services should be established on a case by case basis to suit the needs of individual mothers.

Findings from both the questionnaire given to feeding services and the mothers’ interviews highlight the disparity in pandemic-related service provision. Whilst the low response rate raises caution over the results’ generalisability, it is interesting to note the level of variation across even a small number of services. A larger, UK-wide survey of hospital and community feeding support services would be required to better understand the wider patterns of change throughout the pandemic as well as both short- and long-term consequences of these. Furthermore, inconsistency in availability and quality of feeding support services as well as mothers’ access to it had been commonly reported amongst the literature pre-pandemic (Cartwright and Boath, 2018). As a result, it is difficult to estimate to what extent this gap in the services is related to the pandemic.

Better education of health professionals is needed because addressing health care staff assumptions about breastfeeding abilities with adequate training has been shown to dramatically increase breastfeeding rates (Barbas and Kelleher 2004). Moreover, a sense of encouragement and positivity about their infant’s feeding is invaluable for mothers and can help them to persevere when encountering feeding difficulties. Health professionals should also be encouraged to ask questions and be willing to say when they are unsure about something, so that this may be used as an opportunity to develop understanding. Shying away from discussing Down syndrome out of fear of saying the wrong thing can have negative consequences.

The findings highlight the need for a clearly defined, universal care pathway to reduce inequalities in service access and ensure all mothers of infants with Down syndrome have access to the same level of support. For example, referrals to services should be done automatically when mothers and infants leave hospital, and the services that children may need to access should be clearly set out to the parents early in the child’s life to prevent parents feeling overwhelmed and underinformed. Furthermore, the availability of face-to-face support should be seen as an urgent priority for the wellbeing of new mothers of infants with Down syndrome.
